# Evaluating ChatGPT 4.0 as a Tool for Nuclear Medicine Board Preparation

**DOI:** 10.7759/cureus.92721

**Published:** 2025-09-19

**Authors:** Pierce Herrmann, Kayvon Yazdanbakhsh, Golnaz Lotfian, Keyur Parekh, Sumeet Virmani, Alex Tegeler, Pokhraj P Suthar

**Affiliations:** 1 Department of Diagnostic Radiology and Nuclear Medicine, Rush University Medical Center, Chicago, USA

**Keywords:** ai, chatgpt, ml, nlp, proprietary nlp

## Abstract

Due to their potential use in medical education, large language models (LLMs), a type of generative artificial intelligence (AI), have become increasingly popular. The accuracy of ChatGPT 4.0 (OpenAI, San Francisco, CA) in responding to multiple-choice questions from a standardized board preparation resource for nuclear medicine certification examinations is assessed in this study. A total of 115 text-based questions from 12 chapters were chosen in total; image-dependent questions were not included because of ChatGPT's restrictions on text-only input. Section-by-section and overall accuracy were calculated by comparing the model's replies to the official answer key. ChatGPT performed the worst in pediatric nuclear medicine (75%), while achieving a total accuracy of 86.95%. It received perfect marks in nuclear cardiology and radiopharmacy. Interestingly, model performance did not correlate with the quantity of questions per chapter. According to these results, ChatGPT might be a useful addition to radiology education; nonetheless, topic-level performance variations and opaque reasoning underscore the need for more research prior to wider educational integration.

## Introduction

Large language models (LLMs), in particular, have become increasingly popular across a wide range of industries due to advancements in artificial intelligence (AI). With hundreds of millions of users worldwide, ChatGPT (OpenAI, San Francisco, CA), which was made available to the public in November 2022, has emerged as one of the most popular LLMs [1-3]. Interest in its use in healthcare and medical education has grown due to its accessibility and capacity to produce logical, contextually relevant solutions [4-6].

Because radiology relies on both image-based and text-based interpretation, it is particularly well-suited for LLM integration. Although previous research has examined the usefulness of LLM in clinical support and summary tasks, little is known about how well they perform on standardized board review content [7,8]. To date, only limited studies have evaluated LLM performance on medical board-style examinations, and none have explicitly focused on nuclear medicine question banks. This study, therefore, addresses an important and largely unexplored area of LLM application in medical education.

The accuracy of ChatGPT 4.0 in responding to board-style multiple-choice questions from Nuclear Medicine: A Core Review by Shah et al. [9] is examined in this study. The objective is to assess the validity of ChatGPT in the context of radiology education and investigate its potential as an additional aid for board preparation.

## Materials and methods

This study was designed as a cross-sectional diagnostic accuracy study to evaluate ChatGPT's performance on text-based nuclear medicine board review questions. A total of 115 multiple-choice questions were selected from Nuclear Medicine: A Core Review by Shah et al. [9], spanning all 12 chapters. The chosen items included a mix of three-, four-, and five-option multiple-choice questions, consistent with the original textbook format. To ensure balanced representation and reduce selection bias, we included the first 15 text-based questions per chapter, when available. Although the order of questions in the textbook is not randomized, we selected the first 15 text-based items per chapter to provide a consistent, reproducible sampling frame; potential differences in difficulty between early and later items are acknowledged as a study limitation. For shorter chapters, all non-image-based questions were used. This strategy provided a standardized and reproducible sampling framework across the breadth of topics and was conducted between March and April 2025.

Image-dependent questions were excluded to minimize confounding from the model's image interpretation limitations. Although the publicly available March 2024 version of ChatGPT-4.0 (OpenAI) possesses multimodal functionality, this study deliberately focused on text-only performance to isolate its language reasoning and recall capabilities.

Each question was entered into the ChatGPT interface independently using a uniform prompt: "What is the correct answer to the following multiple-choice question?" To avoid context contamination, no prior interactions were visible to the model, and each query was treated as a separate session, and only a single attempt per question was recorded.

Responses, including both the selected answer and explanatory text, were recorded verbatim. Three board-certified radiologists independently validated ChatGPT's reactions against the textbook answer key. Reviewers were blinded to each other's assessments, and discrepancies were resolved by consensus.

Data were entered into Microsoft Excel (Microsoft Corp., Redmond, WA) and organized by chapter and content domain. Accuracy was calculated at both the chapter level and for the entire dataset. Excluded questions were tracked separately to document the final denominator.

The study was conducted and reported in accordance with the Standards for Reporting Diagnostic Accuracy Studies (STARD) guidelines, ensuring transparency and reproducibility.

**Figure 1:**
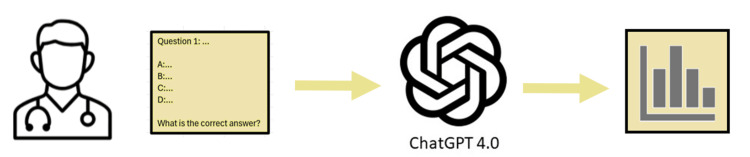
Illustration of provided prompt and associated ChatGPT responses The questions and their associated answers were sourced from the textbook Nuclear Medicine: A Core Review by Shah et al. [9]. Image credit: Golnaz Lotfian

Figure 1 outlines the overall input and validation workflow.

## Results

**Figure 2:**
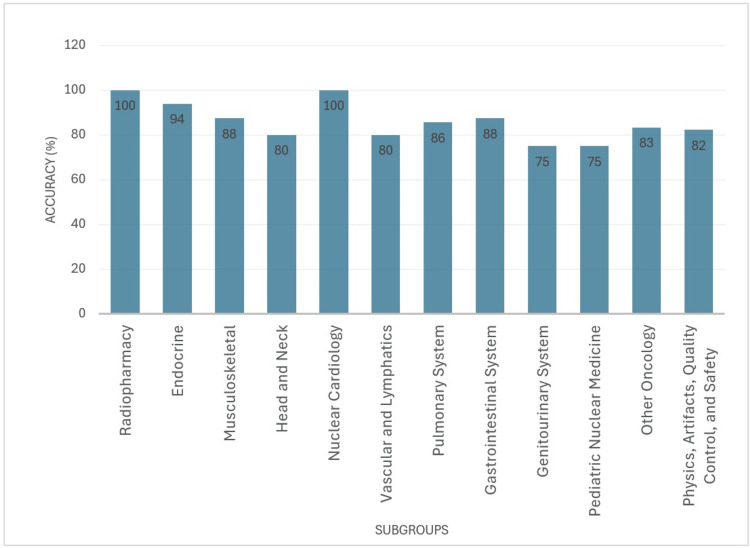
ChatGPT's performance across all subgroups ChatGPT achieved an overall accuracy of 86.95%, with a standard deviation of 7.3 across all subgroups. Notably, it performed perfectly in two subgroups of radiopharmacy and nuclear cardiology.

**Figure 3:**
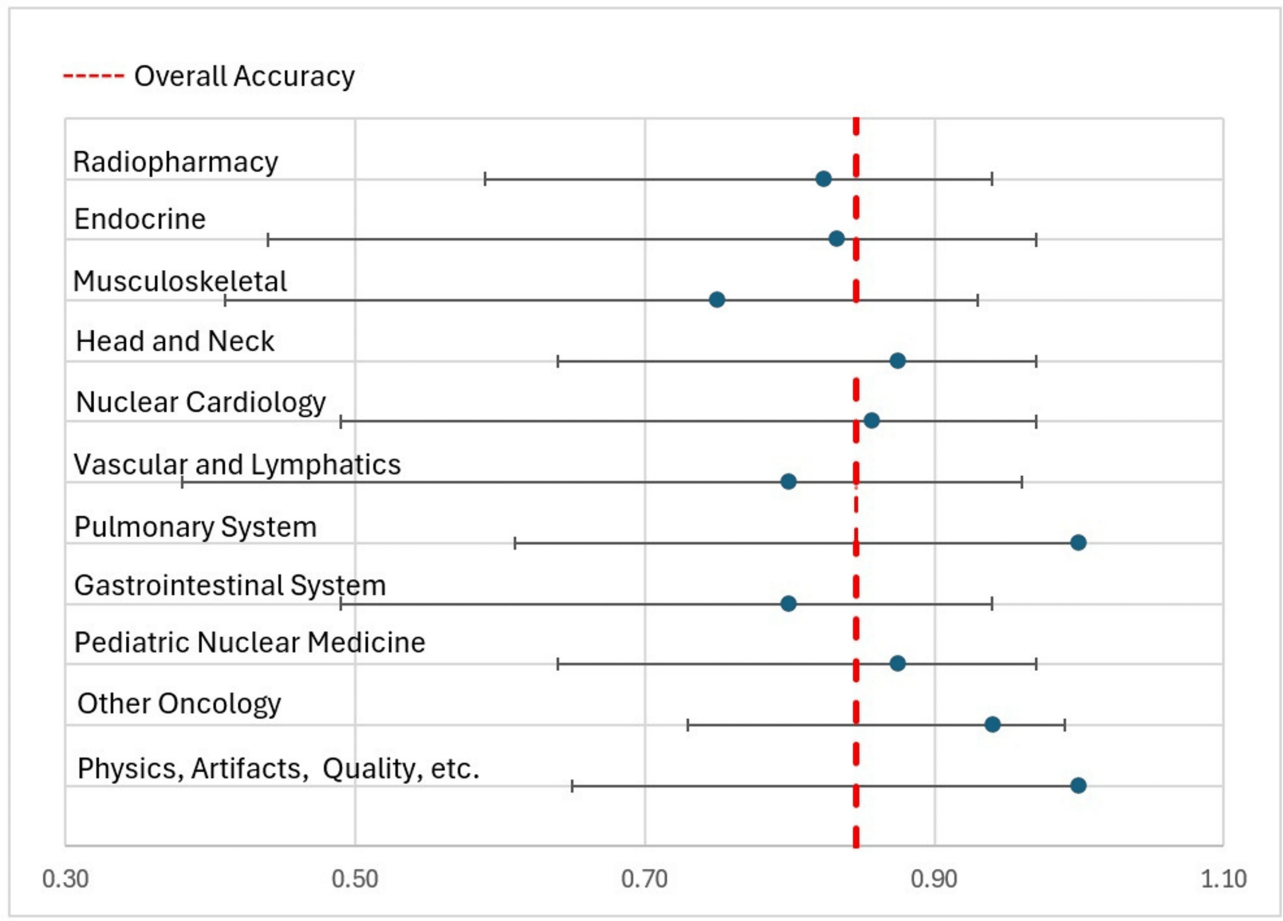
Forest plot across all selected subgroups Forest plot (accuracy with 95% confidence interval) showing individual subgroup accuracy versus baseline (overall accuracy).

Our study found that ChatGPT's performance on nuclear medicine multiple-choice practice questions varied depending on the subject of the question bank, with its accuracy ranging from 75% to 100%. The mean ChatGPT correct score was 86.95%, with a median of 85.55% and a standard deviation of 7.28% (Figure 2 and Figure 3).

**Figure 4:**
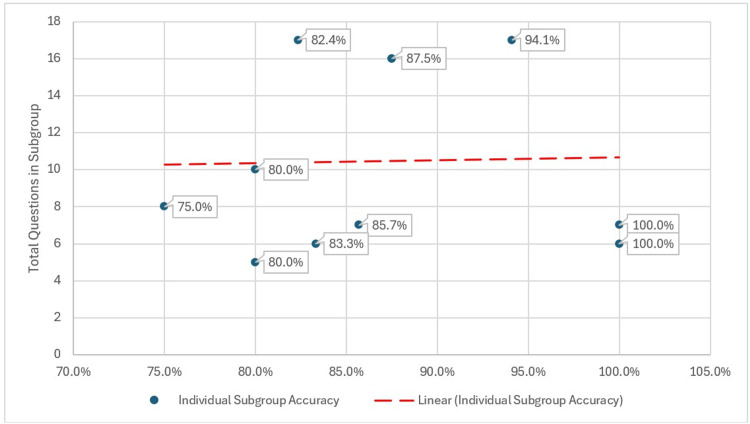
Lack of correlation of accuracy versus total questions available in each subgroup

ChatGPT scored a perfect score in the radiopharmacy and nuclear cardiology sections, correctly answering all seven questions in the former and all six questions in the latter. Given the limited number of items in these sections, these perfect scores should be interpreted with caution, as they may not represent performance on a larger sample of questions. These chapters are outliers for ChatGPT, as they are the only sections with a score more than one standard deviation from the mean of its performance. ChatGPT scored the worst on pediatric nuclear medicine, another outlier, as it was the only score more than one standard deviation below the mean, at 75%, missing two out of eight questions. We hypothesized that, assuming ChatGPT has access to equal amounts of information regarding every chapter, the probability of getting a better score would correlate with the number of questions per section. However, we did not find this to be the case. As seen in Figure 3, there was no correlation between the percentage of correct answers and the total number of questions in that section, as seen in Figure 4.

## Discussion

There has been a rapid surge in advancements in artificial intelligence, particularly in the development and deployment of large language models (LLMs). ChatGPT, developed by OpenAI and released in 2022, represents one of the most widely adopted and capable LLMs available today. Its accessibility and broad application across industries have driven interest in its potential use in medicine, especially within radiology, where interpreting complex, structured information is central to both education and clinical care [10,11].

The potential application of LLMs in radiology is significant. These models have demonstrated the ability to rapidly process text-based inputs, offering potential benefits in streamlining workflows, supporting decision-making, stratifying clinician tasks, and enhancing diagnostic capabilities [8,12]. Our study focused specifically on evaluating ChatGPT's performance in nuclear medicine board-style multiple-choice questions, offering insight into how such tools might be leveraged in medical education.

ChatGPT 4.0 demonstrated strong overall performance, achieving an average accuracy of 86.95% in answering board-style nuclear medicine questions. However, the results revealed variability across topic categories. The model performed flawlessly in specific domains, such as radiopharmacy and nuclear cardiology, but struggled in others, including pediatric nuclear medicine. One hypothesis is that performance may be influenced by the volume of publicly available training data for specific topics. However, our study did not assess training data composition directly, so this theory remains speculative and warrants further investigation. We also found no correlation between the number of questions per chapter and accuracy, suggesting that other factors, such as question complexity, information density, or context, may contribute to this variability. Prior research has proposed that performance in LLMs can fluctuate based on the diagnostic clues provided in each question, a variable not assessed in our study but worth exploring in future work [13].

Model reliability and interpretability

Although ChatGPT demonstrated high accuracy in this assessment, consistency and reliability remain challenges for educational or diagnostic applications. The model's responses are not deterministic; repeating the same prompt may yield slightly different answers, particularly for nuanced or context-heavy questions. This variability, while acceptable in general use, raises concerns in medical settings where reproducibility and precision are critical [11,14].

Another concern is the model's lack of transparent reasoning [12,15]. ChatGPT provides direct answers without citing references or explaining its rationale, limiting its utility as a teaching tool. In medical education, the ability to follow the logic behind a conclusion is essential for learners to develop critical thinking skills. Without insight into how a model arrives at its answer, users may accept incorrect or oversimplified responses at face value, potentially reinforcing misconceptions [15].

Study limitations and model scope

There are various restrictions on this study. First, although image-based questions are essential to the profession, the analysis was limited to questions from a single source: Nuclear Medicine: A Core Review. Therefore, the assessment does not fully represent the range of nuclear medicine board examinations; instead, it only evaluates ChatGPT's performance on text-based knowledge. Although this was necessary because ChatGPT cannot currently understand images, it restricts the extent to which the results can be utilized. In addition, some subspecialty domains contained relatively few questions; thus, perfect scores in these areas should be interpreted cautiously and may not reflect performance across a larger item pool.

Furthermore, we did not examine the effects of clinical vignettes, question structure, or distractions on performance elements that are increasingly recognized as crucial in assessing LLM behavior [16]. To evaluate how effectively the model handles clinical logic versus factual recall, future research should examine these factors more closely, possibly utilizing larger and more diverse datasets. While we hypothesized that topic representation within publicly available training data could influence performance, our study did not directly assess model training data composition; this remains speculative and warrants further investigation.

Lastly, ChatGPT is a general-purpose language model that has not been specially adjusted for medical board examinations or nuclear medicine. It nevertheless had outstanding results in some categories. Its non-transparent and non-domain-specific training data, however, raise concerns over the repeatability of its performance in increasingly complex or specialized diagnostic settings [16].

## Conclusions

ChatGPT 4.0 demonstrated strong overall accuracy (86.95%) in answering nuclear medicine board review questions, with perfect scores in radiopharmacy and nuclear cardiology, and its weakest performance in pediatric nuclear medicine. These results highlight both the promise of AI as a supplemental educational tool and the variability in performance across subspecialty domains, potentially reflecting gaps in topic representation within the model's training data. Notably, no correlation was observed between chapter size and performance, suggesting that factors such as question complexity, answer framing, or prevalence of conditions may play a greater role.

While these findings support the potential role of ChatGPT in radiology education and board preparation, the variability underscores the continued importance of human expertise. AI can serve as a valuable adjunct to enhance comprehension and engagement, but it cannot replace the judgment, experience, and critical reasoning of trained physicians. Future studies should further investigate the factors that influence AI accuracy and explore strategies for integrating these tools responsibly into medical training.
